# Role of preoperative prediction of microvascular invasion in hepatocellular carcinoma based on the texture of FDG PET image: A comparison of quantitative metabolic parameters and MRI

**DOI:** 10.3389/fphys.2022.928969

**Published:** 2022-08-12

**Authors:** Huazheng Shi, Ying Duan, Jie Shi, Wenrui Zhang, Weiran Liu, Bixia Shen, Fufu Liu, Xin Mei, Xiaoxiao Li, Zheng Yuan

**Affiliations:** ^1^ Shanghai Universal Cloud Medical Imaging Diagnostic Center, Shanghai, China; ^2^ Department of Hepatic Surgery VI, Eastern Hepatobiliary Surgery Hospital, The Second Military Medical University, Shanghai, China; ^3^ Department of Radiology, Shanghai Ninth People’s Hospital, Shanghai Jiao Tong University School of Medicine, Shanghai, China

**Keywords:** hepatocellular carcinoma, microvascular invasion, fluorine-18 fluorodeoxyglucose-positron emission tomography (^18^F-FDG-PET), magnetic resonance imaging, texture feature

## Abstract

**Objective:** To investigate the role of prediction microvascular invasion (mVI) in hepatocellular carcinoma (HCC) by ^18^F-FDG PET image texture analysis and hybrid criteria combining PET/CT and multi-parameter MRI.

**Materials and methods:** Ninety-seven patients with HCC who received the examinations of MRI and ^18^F-FDG PET/CT were retrospectively included in this study and were randomized into training and testing cohorts. The lesion image texture features of ^18^F-FDG PET were extracted using MaZda software. The optimal predictive texture features of mVI were selected, and the classification procedure was conducted. The predictive performance of mVI by radiomics classier in training and testing cohorts was respectively recorded. Next, the hybrid model was developed by integrating the ^18^F-FDG PET image texture, metabolic parameters, and MRI parameters to predict mVI through logistic regression. Furthermore, the diagnostic performance of each time was recorded.

**Results:** The ^18^F-FDG PET image radiomics classier showed good predicted performance in both training and testing cohorts to discriminate HCC with/without mVI, with an AUC of 0.917 (95% CI: 0.824–0.970) and 0.771 (95% CI: 0.578, 0.905). The hybrid model, which combines radiomics classier, SUVmax, ADC, hypovascular arterial phase enhancement pattern on contrast-enhanced MRI, and non-smooth tumor margin, also yielded better predictive performance with an AUC of 0.996 (95% CI: 0.939, 1.000) and 0.953 (95% CI: 0.883, 1.000). The differences in AUCs between radiomics classier and hybrid classier were significant in both training and testing cohorts (DeLong test, both *p* < 0.05).

**Conclusion:** The radiomics classier based on ^18^F-FDG PET image texture and the hybrid classier incorporating ^18^F-FDG PET/CT and MRI yielded good predictive performance, which might provide a precise prediction of HCC mVI preoperatively.

## 1 Introduction

Hepatocellular carcinoma (HCC) is one of the most common liver malignancies and one of the leading causes of cancer death in the world (Sung et al., 2021). The main cause of unsatisfactory HCC prognosis is that it is difficult to detect at the early stage because of poor symptoms. The primary curative treatment modality of HCC is partial hepatectomy. However, upon detection, most HCC patients are unsuitable for hepatectomy because of the underlying liver cirrhosis and hepatic dysfunction. Even though HCC patients receive curative surgical resection, there is still a relatively high recurrence rate, even in HCC patients receiving liver transplantation, as high as 15%–30% (Hoffman and Mehta, 2021; Tampaki et al., 2021). Therefore, early diagnosis and accurate prediction of postoperative HCC recurrence are important. Some established factors such as tumor grade, stage, size, liver function, and treatment acted as predictors of postoperative HCC recurrence (Ochiai et al., 2012).

Vascular invasion of HCC representing invasive tumor behavior is a significant predictor of poor outcomes (Jonas et al., 2001). Macrovascular invasion (MVI) could be readily detected by contrast-enhanced CT/MR imaging before surgical resection (Teefey et al., 2003). However, as a histologic finding, microvascular invasion (mVI) is usually visible only on microscopy by histopathology of the surgical specimen, which is difficult to diagnose before surgical resection. Therefore, it is essential to detect clinical predictors to suggest the presence of mVI preoperatively.

Previous research has reported that the status of mVI can be predicted by key imaging and laboratory tests. Several previous studies have reported that tumor margin, capsule, and peritumoral enhancement on CT/MRI scans were significantly associated with mVI (Nishie et al., 2008; Witjes et al., 2012). However, contradictory results were also reported in some studies (Kim et al., 2009; Chou et al., 2014). In clinical practice, developing a reliable preoperative predictor for mVI is still necessary. As a functional molecular imaging modality, fluorine-18 fluorodeoxyglucose (^18^F-FDG) positron emission tomography (PET)/computed tomography (CT) is useful for evaluating HCC differentiation grade by estimating the glucose metabolism of tumor cells (Cho et al., 2017). Recently, several studies have reported the role of ^18^F-FDG PET/CT in defining mVI in patients with HCC. However, there is no consistent conclusion, and the current results showed a wide range of sensitivity, specificity, and accuracy for preoperatively detecting mVI, which indicated that the current PET/CT technique is insufficient alone for establishing a risk factor for mVI (Kornberg et al., 2009; Lee et al., 2009; Lin et al., 2017; Kim and Kim, 2021). Recently, Li et al. conducted radiomics analysis on ^18^F-FDG PET/CT to preoperatively predict mVI and prognosis in patients with very early and early stages of HCC, which is the importance of precise treatment of patients (Li et al., 2021).

In this retrospective study, we aimed to verify the comprehensive value of ^18^F-FDG-PET/CT in the prediction of mVI by quantitative uptake measurement and image texture analysis. We also focus on the role of the hybrid model of incorporating ^18^F-FDG-PET/CT and multi-parameter MRI. We hypothesized if ^18^F-FDG-PET/CT findings predict mVI and, more importantly, the added value, if any, of PET/CT for the hybrid model in the prediction of mVI.

## 2 Materials and methods

### 2.1 Patients

This retrospective study was conducted in accordance with the Declaration of Helsinki proposed in 1975 and revised in 2000 and was approved by the Ethics Committee of the universal medical imaging center, Shanghai University (SHQJ-2019-05). The consecutive HCC patients were confirmed by histopathology after partial hepatectomy from January 2018 to April 2021. The inclusion criteria were as follows: (Sung et al., 2021) age >18 years; (Hoffman and Mehta, 2021) primary HCC confirmed by pathology of surgical specimens; (Tampaki et al., 2021) multi-parameter MR images containing conventional unenhanced MR imaging (including T1WI and T2WI), dynamic contrast-enhanced T1WI (including the arterial phase imaging, portal venous phase imaging, and delayed phase imaging), and diffusion-weighted imaging (DWI) (with *b*-value of 0 and 800 s/mm^2^); (Ochiai et al., 2012) PET/CT and multi-parameter MRI examination approximately within 4 weeks before surgery; and (Jonas et al., 2001) no history of preoperative anti-cancer treatment. The exclusion criteria of this study were as follows: (Sung et al., 2021) preoperative images showing macrovascular invasion; (Hoffman and Mehta, 2021) HCC patients who underwent any anti-cancer treatment before partial hepatectomy; (Tampaki et al., 2021) those with time intervals of PET/CT, MRI, and surgery more than 4 weeks; (Ochiai et al., 2012) those with no pathology slides available for review; and (Jonas et al., 2001) images with artifacts affecting evaluation.

The patients enrolled in this study were randomly divided into two cohorts (training and testing) with a ratio of 7:3 using computer-generated random numbers.

### 2.2 ^18^F-FDG PET/CT acquisition and image analysis

#### 2.2.1 ^18^F-FDG PET/CT acquisition

This study examined all ^18^F-FDG PET/CT acquisitions with the SIEMENS Biograph mCT Flow PET/CT system (Siemens Medical Solutions United States, Inc.). After at least 6 h of fasting, the patient was intravenously administered a standard dose (3.7 MBq/kg) of ^18^F-FDG, followed by image acquisition 60 min later, from the thigh to the head. Whole-body non-contrast enhancement CT scanning protocols were as follows: 120 kVp, 30–170 mAs adjusted to the patient’s body weight and with a section width of 3 mm and collimation of 0.75 mm. An emission scan was performed in a three-dimensional (3D) mode with an acquisition time of 1.7 min per bed position. PET images were reconstructed by a 2-iteration, 21-ordered-subset expectation maximization algorithm using CT images for attenuation correction.

#### 2.2.2 ^18^F-FDG PET metabolic and volumetric parameters

Standardized uptake values (SUV) were calculated by the region-of-interest (ROI) technique. In order to calculate SUVmax and SUVmean, manually defined circular ROI was drawn on attenuation-corrected emission images selected for the largest axial image of the HCC lesion. On the PET image, an ellipse iso-contour was drawn covering the lesion, and the volume of interest (VOI) in 3D, that is, metabolic tumor volume (MTV), was obtained semi-automatedly with an iso-contour SUV value threshold of 2.5 (Kim et al., 2017). Total lesion glycolysis (TLG) was calculated by multiplying the selected PET volume by the average SUV within that volume: TLG = MTV × (average SUV). If the lesion had a low uptake of ^18^F-FDG, the VOI was calculated on CT images and was then copied to PET to obtain the VOI on PET. Contrast-enhanced MRI was sometimes used to help delineate lesions.

The parameters of HCC ^18^F-FDG metabolic avidity SUVmax, SUVmean, the ratio of the maximum standardized uptake value of tumor to the average standardized uptake value of normal liver (TLRmax), and the average tumor-to-normal liver standardized uptake value ratio (TLRmean) were calculated and recorded.

#### 2.2.3 Texture analysis on axial ^18^F-FDG PET images

##### 2.2.3.1 Data standardization

Before texture analysis of the ^18^F-FDG PET image, the data standardization procession to minimize the influence of image contrast and brightness variations was performed by adopting a method of normalizing the intensities of greyscale images into the range of mean value ± three standard deviations (SD) (μ − 3SD, μ + 3SD).

##### 2.2.3.2 ^18^F-FDG PET image texture analysis

**FIGURE 1:**
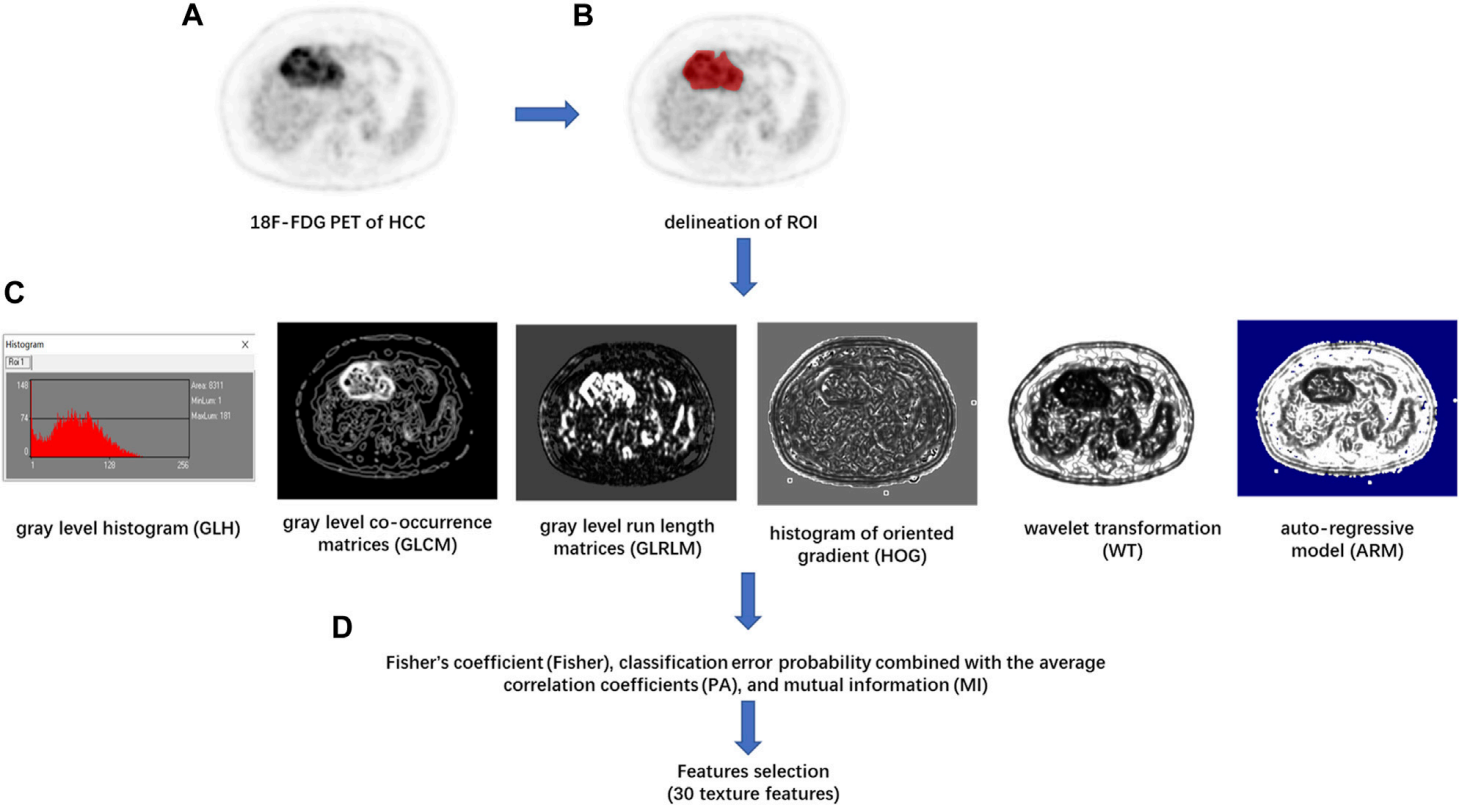
Flowchart of texture features (TF) extraction. **(A)** The ^18^F-FDG PET image of the maximum axial section of hepatocellular carcinoma (HCC) was chosen. **(B)** The region of interest (ROI) of the tumor was drawn in red on MaZda. **(C)** The gray level histogram (GLH), gray level co-occurrence matrices (GLCM), gray level run length matrices (GLRLM), histogram of oriented gradient (HOG), wavelet transformation (WT), and the autoregressive model (ARM) of tumor were calculated, respectively. **(D)** A combination of feature selection algorithms, including Fisher’s coefficient (Fisher), classification error probability combined with the average correlation coefficients (PA), and mutual information (MI), was used to determine 30 texture features with the highest discriminative power for differentiation HCC with or without between mVI.

Texture feature extraction and selection were performed with the MaZda software package (version 4.6, available at http://www.eletel.p.lodz.pl/mazda/) (Szczypinski et al., 2009). The largest axial ^18^F-FDG PET image of each HCC lesion was selected for image texture analysis. The ROI was manually circumscribed over the entire HCC lesion possibly on each selected image by an experienced radiologist (Figure 1). MaZda software allows the computation of almost 300 texture features based on the image histogram, co-occurrence matrix (COM), run-length matrix, absolute gradient, auto-regressive model, and wavelet transforms (WAV) (Pinker et al., 2018). These texture features (Supplementary Table S1) were extracted from each ROI.

In order to achieve the highest differentiation power of HCC with or without mVI, avoid the problem of dimensionality, and reduce the bias, the image texture feature selection was performed by a feature selection algorithm combining Fisher’s coefficient (Fisher), classification error probability combined with the average correlation coefficients (PA), and mutual information (MI) on module B11. Then, 30 texture features with the highest discriminative power were selected. In the training cohort, the classification procedures were conducted using principal component analysis (PCA), linear discriminant analysis (LDA), and non-linear discriminant analysis (NDA) on module B11 of the MaZda software package, and the sensitivity, specificity, positive predictive value, negative predictive value, and accuracy of diagnosis were calculated. The best classification procedure was selected as the radiomic classifier with the highest accuracy. In the testing cohort, the optimal 30 texture features were selected according to the result of the training cohort, and the predictive performance of the radiomic classifier for differentiation of the status of mVI of HCC was calculated.

### 2.3 MRI technique and image analysis

All MRI examinations were performed with a 3.0-T MRI scanner (Magnetom Aera, Siemens Healthcare, Erlangen, Germany; or Ingenia, Philips Healthcare, Best, the Netherlands). The imaging protocol was as follows: axial fast spin echo T2WI with fat saturation using a navigator-triggered technique, DWI using a single-shot echoplanar imaging pulse sequence with *b*-values of 0 and 800 s/mm^2^. The apparent diffusion coefficient (ADC) of the HCC lesion was measured on the ADC map by the ROI drawn by two radiologists circumscribing the entire lesion on the largest axial image in consensus. T1WI images were obtained before and after administration of gadolinium injection solution, with a dose of 0.1 mmol per kilogram of body weight and an injection rate of 2 ml/sec. The arterial phase (AP) images were acquired approximately 25 s after contrast material injection. The portal venous phase (PVP) images were acquired approximately 55–65 s after the start of contrast material administration, and delayed phase (DP) images were acquired 90–100 s after contrast material injection. All parameters for the MRI sequences are summarized in Supplementary Table S2.

#### 2.3.1 Image analysis

MR images were retrospectively analyzed on a workstation or a picture archiving and communication system (PACS). Two clinically experienced radiologists evaluated the MR images in consensus to obtain reliable results. Both readers were blinded to the status of mVI.

The two radiologists qualitatively made the following: 1) classified the arterial phase enhancement patterns on dynamic contrast-enhanced MRI into three patterns: hypervascular HCC, isovascular HCC, and hypovascular HCC; (Hoffman and Mehta, 2021) determined the presence or absence of intratumoral artery; 2) classified the patterns of tumor margin into two patterns: smooth margin or non-smooth margin; and 3) determined HCC with or without peritumoral enhancement on the AP images.

**FIGURE 2:**
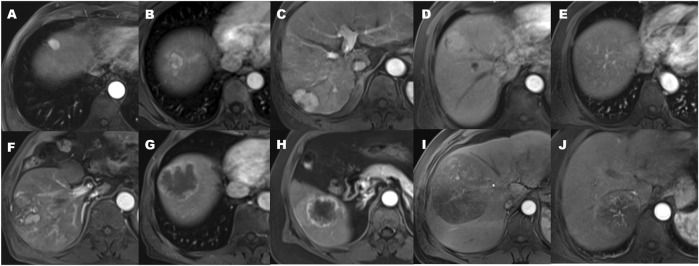
The patterns of arterial phase enhancement of hepatocellular carcinoma on contrast-enhanced MRI. **(A)** Homogeneously hypervascular HCC. **(B)** Hypervascular HCC with slit-like hypovascular foci. **(C)** With multifocal hypovascular foci. **(D)** Hypervascular HCC with the peripheral hypovascular area. **(E)** Completely isovascular HCC. **(F)** HCC with a nodular or irregular-shaped hypovascular portion at the inner area. **(G)** Hypovascular HCC with irregular rim-like enhancement. **(H)** Hypovascular HCC with the peripheral hypervascular area, discontinuous rim or crescent-like, and the presence of peritumoral enhancement seen in the arterial phase. **(I)** Hypovascular HCC with linear or spot-like hypervascular foci. **(J)** the presence of an intratumoral artery.

**FIGURE 3:**
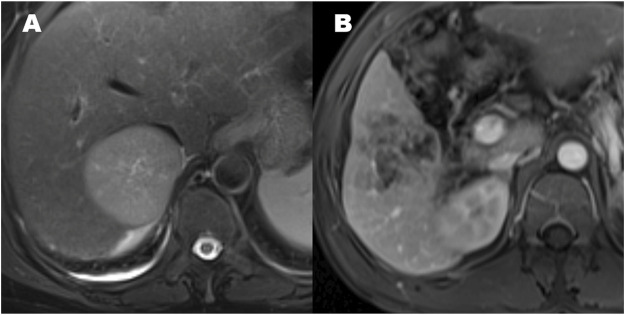
The patterns of hepatocellular carcinoma tumor margin. **(A)** Smooth tumor margin and intact tumor capsule. **(B)** Tumor with a non-smooth margin.

Qualitative findings on dynamic contrast-enhanced MR imaging were defined as follows: 1) hypervascular HCC: homogeneously hypervascular, hypervascular with slit-like hypovascular foci, or multifocal hypovascular foci, with a peripheral hypovascular area (Figure 2); 2) hypovascular HCC: with a nodular- or irregular-shaped hypointense portion at an inner area, with irregular rim-like enhancement, with a peripheral hypervascular area, discontinuous rim, or crescent-like, with linear or spot-like hypervascular foci, or completely hypovascular HCC (Figure 2) (Rhee et al., 2021); 3) intratumoral artery: the blood vessels within the tumor in AP images (Figure 2) (Segal et al., 2007); 4) a smooth margin defined as a nodular-shaped tumor without extranodular extension or infiltrative, non-smooth margins defined as a nodule with extranodular extension or an infiltrative margin (Figure 3) (Nakashima et al., 2003; Segal et al., 2007); and 5) peritumoural enhancement on AP images: defined as a patchy hyperintense area adjacent to the tumor with broad contact to the tumor border on the AP images, presenting isointense to liver parenchyma on the DP images (Figure 2) (Kim et al., 2009).

### 2.4 Intra-observer and inter-observer agreement

The reproducibility of the intra-observer and inter-observer agreement for texture analysis was measured using 20 randomly chosen samples drawn from axial ^18^F-FDG PET images and ADC map by two radiologists blinded from patients’ characteristics. To evaluate intra-observer reproducibility, the first radiologist delineated an ROI twice within 2 weeks following the same procedure. Meanwhile, the second radiologist also independently delineated the ROI once following the same procedure. Then, the inter-observer agreement was assessed by comparing the results with the texture features extracted calculated from the first ROI delineation by the first radiologist. The intraclass correlation coefficient (ICC) was used to evaluate the intra-observer and inter-observer agreements. An ICC >0.75 indicated satisfactory agreement.

### 2.5 Microvascular invasion evaluation by histopathology

All surgical specimens and the status of mVI were reviewed and evaluated by an experienced pathologist in liver pathology. mVI was defined as the presence of a tumor in a portal vein, hepatic vein, or a large capsular vessel of the surrounding hepatic tissue lined by the endothelium that was visible only on microscopy (Roayaie et al., 2009; Xu et al., 2019). We categorized the HCC patients with mVI as the mVI-positive (mVI+) group and the HCC patients without mVI as the mVI negative (mVI−) group.

### 2.6 Statistical analysis

Inter-reader agreement was expressed by Cohen’s kappa coefficient. A kappa statistic of 0.8–1.0, 0.6–0.79, 0.40–0.59, 0.2–0.39, and 0–0.19 was considered excellent, good, moderate, fair, and poor agreement, respectively.

For categorical variables, the differences between the mVI (−) and mVI (+) groups were analyzed by the Chi-squared test or Fisher’s exact test. For continuous variables with a normal distribution, an independent-samples *t*-test was used to test the significant difference of the mVI (−) and mVI (+) groups; for continuous variables with a skewed distribution, a non-parametric Mann–Whitney *U* test was used. A two-tailed *p*-value less than 0.05 was considered that the difference was statistically significant. SPSS software (SPSS version 24.0; SPSS Inc., Chicago, IL, United States) was used to perform statistical analysis. The predictive value of each factor for mVI was determined by analysis of the area under the ROC curve (AUC). The differences in AUCs were compared by the DeLong test (DeLong et al., 1988) performed using MedCalc software (version 20.023). The calibration of the hybrid model was performed by comparing the predicted and actual probability of mVI by the Hosmer–Lemeshow test.

## 3 Results

For the intra-observer and inter-observer agreement, radiomics features achieved satisfactory consistency. There was no radiomics feature to be eliminated. The mean ICC was 0.956 (range, 0.892 to 1, *p* < 0.001) in the intra-observer agreement and the mean ICC was 0.915 (range, 0.751 to 0.999, *p* < 0.001) in the inter-observer agreement.

**FIGURE 4:**
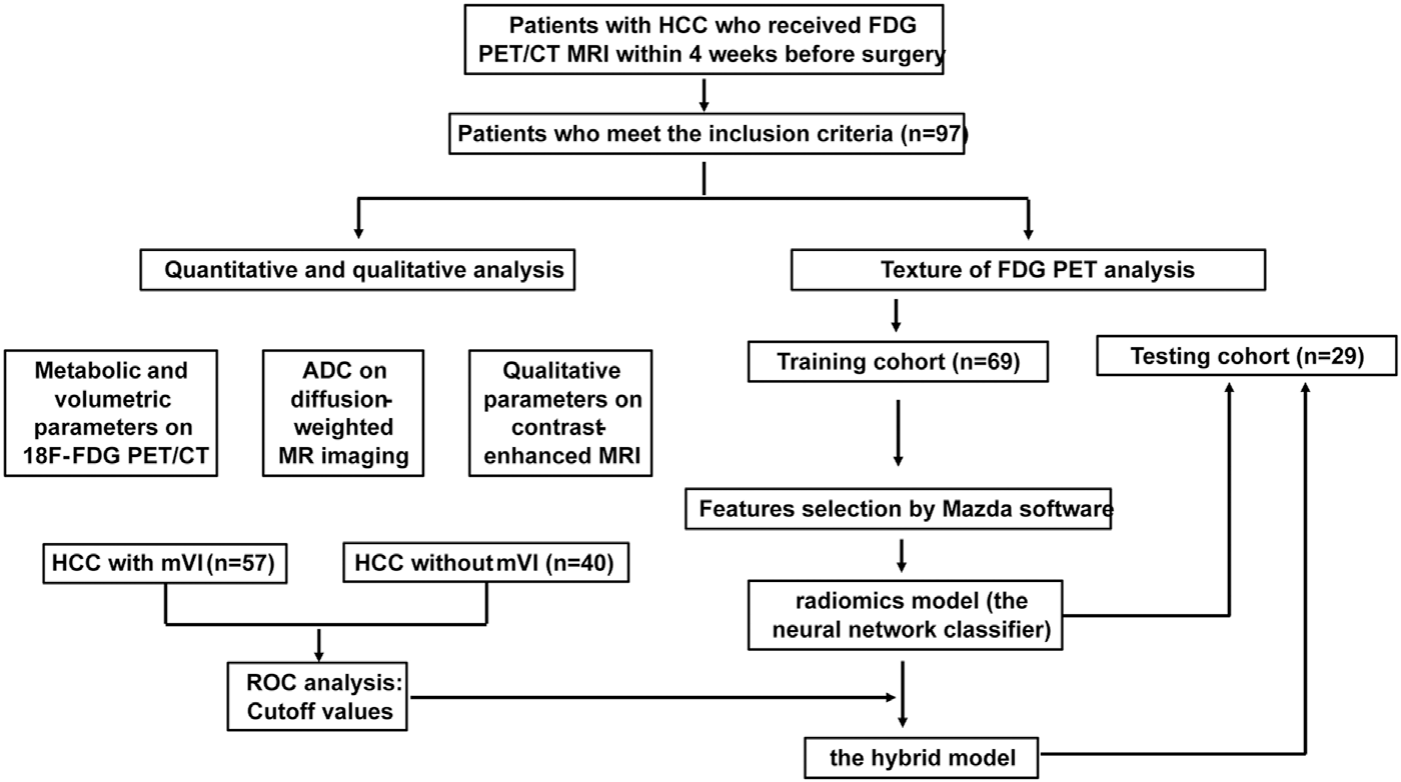
Study flowchart for predicting mVI by analyzing PET texture features and the hybrid model incorporated ^18^F FDG PET/CT and MRI in training and testing set.

In this study, the study flowchart for predicting mVI based on texture features, metabolic and volumetric parameters, and multi-parameter MRI is shown in Figure 4. A total of 97 patients’ clinical characteristics are presented in Table 1.

**TABLE 1:** Clinical characteristics of patients with HCC in the training and testing cohort.

		Training cohort (*n* = 68)			Testing cohort (*n* = 29)			*p*-value***
		mVI+ (*n* = 38) *n* (%) or median (IQR)	mVI− (*n* = 30) *n* (%) or median (IQR)	*p*-value*	mVI+ (*n* = 19) *n* (%) or median (IQR)	mVI− (10) *n* (%) or median (IQR)	*p*-value**	
Sex				0.452			0.414	0.519
	Male	26 (38.2)	23 (33.8)		11 (37.9)	8 (27.6)		
	Female	12 (17.6)	7 (10.3)		8 (27.6)	2 (6.9)		
Age (years)		55 (36.5, 59.3)	55 (50, 63.3)	0.158	50 (50, 62)	46 (55, 62)	0.628	0.512
HBV infection				0.965			0.450	0.583
	Absent	15 (22.1)	12 (17.6)		11 (37.9)	4 (13.8)		
	Present	23 (33.8)	18 (26.5)		8 (27.6)	6 (20.7)		
Child–Pugh				0.973			1.000	0.961
	A	29 (42.6)	23 (33.8)		14 (48.3)	7 (24.1)		
	B	9 (13.2)	7 (10.3)		5 (17.2)	3 (10.3)		
Liver cirrhosis				0.833			0.245	0.420
	Absent	13 (19.1)	11 (16.2)		8 (27.6)	7 (24.1)		
	Present	25 (36.8)	19 (27.9)		11 (37.9)	3 (10.3)		
AFP (ng/ml)				0.357			0.270	0.932
	≤200	16 (23.5)	16 (23.5)		12 (41.4)	4 (13.8)		
	>200	22 (32.3)	14 (20.6)		7 (24.1)	6 (20.7)		
Image tumor size (mm)		42 (18.8, 79.3)	40 (17.5, 69.8)	0.923	64.0 (55.0, 85.0)	67.5 (44.0, 96.3)	0.982	0.888

HCC, hepatocellular carcinoma; HBV, hepatitis B virus; AFP, alpha-fetoprotein; mVI, microvascular invasion; IQR, interquartile range.

*The difference in HCC with mVI and without mVI in the training cohort.

**The difference in HCC with mVI and without mVI in the testing cohort.

***The difference in HCC with mVI and without mVI in the pooled cohort.

Of the 97 patients with HCC, 58.8% (57/97) had tumors with mVI and 41.2% (40/97) without mVI. In the training cohort, mVI (+) was presented in 55.9% (38/68) of tumors, similar to 65.5% (19/29) seen in the testing cohort (*p* > 0.05). There were also no significant differences in other baseline clinical features between the training and testing groups (all *p* > 0.05, Table 1), which indicated that the distribution of baseline clinical-pathologic characteristics in the training and testing group was balanced.

**FIGURE 5:**
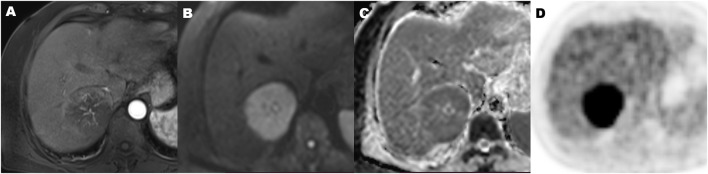
A 70-year-old hepatocellular carcinoma in liver segment VII woman with microvascular invasion, demonstrating Hypovascular enhancement pattern, high metabolic of SUVmax 9.2, MTV 134.2 cm^3^, TLG 915.2, TLR 4.5, and ADC of 0.86 × 10^−3^ mm^2^/s. Contrast-enhanced MR in arterial phase image **(A)**, diffusion-weighted MR image **(B)**, ADC map **(C)**, and ^18^F-FDG PET **(D)** (MTV, metabolic tumor volume; TLG, total lesion glycolysis; TLR, ratio of tumor-to-liver SUV; ADC, apparent diffusion coefficient).

**FIGURE 6:**
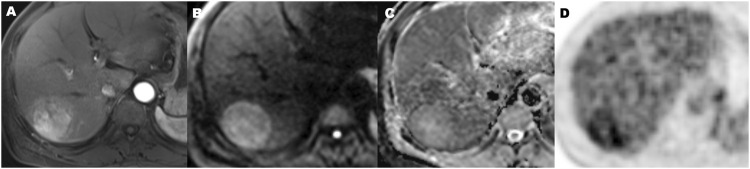
A 56-year-old hepatocellular carcinoma in liver segment VII man without microvascular invasion, demonstrating hypervascular enhancement pattern, low metabolic of SUVmax 3.5, MTV 72.2 cm^3^, TLG 196.5, TLR 1.9, and ADC value of 1.1 × 10^−3^ mm^2^/s. Contrast-enhanced MR in arterial phase image **(A)**, diffusion-weighted MR image **(B)**, ADC map **(C)**, and ^18^F-FDG PET **(D)** (MTV, metabolic tumor volume; TLG, total lesion glycolysis; TLR, ratio of tumor-to-liver SUV; ADC, apparent diffusion coefficient).

In the pooled cohorts, the values of SUVmax, SUVmean, TLRmax, TLRmean, and ADC of HCC with mVI (*n* = 57) were higher than HCC without mVI (*n* = 40) (all *p* < 0.05). There was no significant difference in MTV, TLG, and ADC/ADC_liver between HCC with and without mVI (Table 2; Figures 5, 6).

**TABLE 2:** Radiologic findings of primary HCC in the training and testing cohorts.

	Training cohort (*n* = 68)			Testing cohort (*n* = 29)			*p*-value***
	mVI+ (*n* = 38) *n* (%) or median (IQR)	mVI− (*n* = 30) *n* (%) or median (IQR)	*p* value*	mVI+ (*n* = 19) *n* (%) or median (IQR)	mVI− (10) *n* (%) or median (IQR)	*p*-value**	
SUVmax			0.056			0.068	0.011
	6.2 (3.9, 8.3)	4.0 (2.6, 4.8)		7.8 (4.0, 9.3)	4.5 (2.6, 8.2)		
SUVmean			0.022			0.211	0.006
	4.2 (2.6, 5.6)	3.0 (2.1, 3.3)		4.7 (2.9, 6.6)	3.4 (2.4, 5.8)		
TLRmax			0.006^a^			0.153	0.001^a^
	3.1 (2.3, 4.7)	2.1 (1.7, 2.5)		4.0 (2.0, 6.4)	2.6 (1.5, 4.7)		
TLRmean			0.029^a^			0.338	0.017^a^
	2.4 (1.4, 3.2)	1.6 (1.3, 1.8)		2.5 (1.5, 4.3)	1.8 (1.5, 3.4)		
MTV			0.168			0.737	0.303
	33.0 (6.7,141.3)	15.9 (5.8, 104.9)		101.2 (18.5, 180.8)	81.5 (8.3, 241.2)		
TLG			0.155^a^			0.728	0.102
	115.0 (22.6, 940.2)	48.1 (13.2, 350.4)		661.3 (70.4, 1,507.2)	255.5 (31.4, 1728.2)		
ADC (×10^−3^ mm^2^/s)			0.091			0.066	0.014
	1.29 (1.07, 1.42)	1.12 (0.94, 1.25)		1.19 (0.87, 1.54)	1.03 (0.83, 1.25)		
ADC/ADC_liver			0.365			0.945^a^	0.650^a^
	0.9 (0.8, 1.3)	1.0 (0.9, 1.2)		1.3 (1.0, 1.7)	1.1 (1.0, 1.8)		
Arterial phase enhancement pattern		<0.001			0.698	<0.001	
Hypervascular	7 (10.3)	19 (27.9)		6 (20.7)	4 (13.8)		
Hypovascular	31 (45.6)	11 (16.2)		13 (44.8)	6 (20.7)		
Intratumoral artery		1.000			0.372	0.642	
Presence	5 (7.4)	4 (5.9)		3 (10.3)	3 (10.3)		
Absence	33 (48.5)	26 (38.2)		16 (55.2)	7 (24.1)		
Tumor margin		0.053			0.694	0.033	
Smooth	22 (32.4)	24 (35.3)		7 (24.1)	5 (17.2)		
Non-smooth	16 (23.5)	6 (8.8)		12 (41.4)	5 (17.2)		
Peritumoral enhancement in the arterial phase		0.204			0.449	0.077	
Presence	10 (14.7)	5 (7.4)		9 (31.0)	3 (10.3)		
Absence	28 (41.2)	25 (36.8)		10 (34.5)	7 (24.1)		

HCC, hepatocellular carcinoma; mVI, microvascular invasion; IQR, interquartile range; TLRmax, SUVmax/SUVmen_liver; TLRmean, SUVmean/SUVmen_liver; MTV, metabolic tumor volume; TLG, total lesion glycolysis; ADC, apparent diffusion coefficient.

*The difference in HCC with mVI and without mVI in the training cohort.

**The difference in HCC with mVI and without mVI in the testing cohort.

***The difference in HCC with mVI and without mVI in the pooled cohort.

a Non-parametric Mann–Whitney *U* test.

ROC curve analysis showed that the cutoff values of SUVmax, SUVmean, TLRmax, TLRmean, and ADC for predicting HCC with mVI were 5.65, 3.79, 2.53, 1.92, and 1,171.5, respectively, with the largest Youden indexes but moderate diagnostic efficacy (all AUC < 0.70) (Table 3). In all quantitative metabolic parameters, the predictor with the highest diagnostic efficacy is SUVmax with the largest AUC (0.698, 95% CI: 0.593 to 0.803, *p* = 0.001), providing sensitivity and specificity of 59.6% and 80.0% at a cutoff value of 5.65 (Table 4; Supplementary Figure S1).

**TABLE 3:** ROC results of ^18^F-FDG PET/CT parameters and ADC in predicting HCC with mVI in the pooled cohort.

Characteristic parameter	Area under curve	*p*-values	Sensitivity (%)	Specificity (%)	Cutoff
SUVmax	0.698 (0.593, 0.803)	0.001	59.6	80.0	5.65
SUVmean	0.676 (0.567, 0.786)	0.003	66.7	77.5	3.79
TLRmax	0.693 (0.586, 0.799)	0.001	68.4	70.0	2.53
TLRmean	0.643 (0.531, 0.756)	0.017	64.9	75.0	1.92
ADC(×10^−3^mm^2^/s)	0.651 (0.537, 0.765)	0.012	68.4	75.0	1.17

ROC, receiver operating-characteristic curve; HCC, hepatocellular carcinoma; mVI, microvascular invasion; TLRmax, SUVmax/SUVmen_liver; TLRmean, SUVmean/SUVmen_liver; ADC, apparent diffusion coefficient.

**TABLE 4:** Diagnostic performance of hepatocellular carcinoma with microvascular invasion in the training and testing cohorts.

	True positive	False positive	False negative	True negative	Sensitivity	Specificity	Positive-predictive value	Negative-predictive value	Accuracy
Training cohort (*n* = 68)									
Radiomics model	38	5	0	25	100	83.3	88.4	100	92.6%
SUVmax	20	5	18	25	52.6	83.3	80	58.1	66.2
ADC	26	8	12	22	68.4	73.3	76.5	64.7	70.6
Hypovascular	31	11	7	19	81.6	63.3	73.8	73.1	73.5
Non-smooth margin	16	6	22	24	42.1	80	72.7	52.2	58.8
Hybrid model	36	0	2	30	94.7	100	100	93.8	97.1
Testing cohorts (*n* = 29)									
Radiomics model	16	3	3	7	84.2	70	84.2	70	79.3
SUVmax	14	3	5	7	73.7	70	82.4	58.3	72.4
ADC	13	6	2	8	86.7	57.1	68.4	80	72.4
Hypovascular	13	6	6	4	68.4	40	68.4	40	58.6
Non-smooth margin	12	5	7	5	63.2	50	70.6	41.7	58.6
Hybrid model	19	3	1	7	95	70	86.4	87.5	86.7

On dynamic contrast-enhanced MRI review of pooled all cohorts, the HCCs with mVI were significantly associated with enhancement patterns on AP imaging and tumor margin (all *p* < 0.05; Table 2). Most HCCs with mVI (44/57, 77.2%) demonstrated hypovascular lesions (Figure 5). The HCCs with mVI (28/57, 49.1%) manifest as having more frequency of non-smooth tumor margin than the HCCs without mVI (11/40, 27.5%).

Based on the above results, we selected the quantitative parameters of SUVmax and ADC, as well as the qualitative parameters of the hypovascular enhancement pattern on AP MR imaging and non-smooth tumor margin for subsequent analysis.

### 3.1 Training cohort

Based on the feature selection algorithm combining Fisher, PA, and MI coefficients in features modeling, 30 optimal features are selected and listed in Supplementary Table S3.

Compared to PCA (47.1%, 32/68) and LDA (19.1%, 13/68), NDA had the lowest misclassification rate with 7.4% (5/68) for all these three classification procedures by MaZda software (*p* < 0.01). The NDA classification with an AUC of 0.917 (95% CI: 0.824–0.970) showed a sensitivity of 100%, a specificity of 83.3%, a positive predictive value of 88.4%, a negative predictive value of 100%, and an accuracy of 92.6% (Table 4).

Combining radiomics classification results based on the texture of the ^18^F-FDG-PET image, SUVmax, ADC, and the qualitative parameters of the hypovascular arterial phase enhancement pattern and non-smooth tumor margin, the hybrid model regression equation was as follows:

Logit (P) = −2.077 + 0.203 × SUVmax + 2.825 × ADC − 4.717 × Radiomics − 0.041 × non-smooth − 0.664 × hypo-vascular.

*P* is the probability of HCC with mVI. For *p* ≥ 0.5, the lesion was expected to be HCC with mVI, whereas the other lesions were categorized as HCC without mVI.

The hybrid model with an AUC of 0.996 (95% CI: 0.939, 1.000; *p* < 0.001) yielded a sensitivity of 94.7%, a specificity of 100%, a positive predictive value of 100%, a negative predictive value of 93.8%, and an accuracy of 97.1% (Table 4). The difference in AUC between the radiomics classification model and the hybrid model was significant (*p* = 0.017). The calibration of the hybrid model was performed by comparing the predicted and actual probability of mVI by the Hosmer–Lemeshow test (*p* > 0.05). (Supplementary Figure S2A) The difference between the predicted and actual probabilities of mVI showed no statistical significance.

### 3.2 Testing cohort

We selected 30 texture features that were consistent with the training cohort. By using the neural network NDA classifier test included in module B11, the misclassification rate for HCC with *versus* without mVI was 20.7% (6/29). The sensitivity, specificity, positive predictive, negative predictive, and accuracy values were 84.2%, 70.0%, 84.2%, 70.0%, and 79.3%, respectively, with an AUC of 0.771 (95% CI: 0.578, 0.905) (Table 4).

In the testing cohort, the hybrid criteria yielded an AUC of 0.953 (95% CI: 0.883, 1.000; *p* < 0.001), a sensitivity of 95.0%, a specificity of 70%, a positive predictive value of 86.4%, a negative predictive value of 87.5%, and an accuracy of 86.7% (Table 4).

The performances of the radiomics and hybrid models to predict HCC with mVI were also good in the testing cohort, indicating their robustness. The difference in AUC between radiomics criteria and hybrid criteria was also significant (*p* = 0.013), indicating that the hybrid model incorporated ^18^F-FDG PET/CT and MRI yielded better predictive performance. The calibration of the hybrid model was performed by comparing the predicted and actual probability of mVI by the Hosmer–Lemeshow test (*p* > 0.05) (Supplementary Figure S2B). The difference between the predicted and actual probability of mVI showed no statistical significance.

## 4 Discussion

To our knowledge, this study has produced the first texture-based radiomics model of ^18^F FDG PET and a hybrid model that incorporated texture features of ^18^F-FDG PET, quantitative metabolic parameters, and quantitative and qualitative MRI parameters for predicting the status of mVI in HCC. We found that the radiomics model based on the texture of ^18^F-FDG PET had a good diagnostic performance with an AUC of 0.917 (95% CI: 0.836–0.998) and 0.771 (95% CI: 0.578 TO 0.966) in the training and testing cohorts, respectively. Furthermore, hybrid criteria combining ^18^F-FDG PET and MRI could significantly increase diagnostic performance more than the radiomics model (*p* < 0.05) and yield an AUC of 0.996 (95% CI: 0.939, 1.000; *p* < 0.001) and 0.953 (95% CI: 0.883, 1.000) in the training and testing cohorts, respectively. Accordingly, our results may increase the accuracy of preoperative detection of the HCC with or without mVI. It is useful for planning the most appropriate treatment strategy and improving the prognosis of patients with HCC.

Despite progressions in diagnostic and therapeutic modalities, the prognosis of the patient with HCC is still to be improved due to the recurrence rate after treatment remaining high (Mlynarsky et al., 2015). The high heterogeneity of the HCC may have resulted in a varied prognosis (Lu et al., 2016; Lin et al., 2017). As a pathological feature, mVI reflects the invasiveness of the tumor, which usually appears in aggressive HCC while not in low-grade HCC. The patient with HCC presenting mVI has a shorter disease-free survival (DFS) due to a higher risk of tumor recurrence (Miyata et al., 2006). Therefore, mVI is an important prognostic factor of HCC and plays an important role in planning a personalized therapeutic strategy (Erstad and Tanabe, 2019; Xu et al., 2019). It is necessary to detect clinical predictors to suggest the presence of mVI preoperatively in order to establish a personalized therapeutic strategy.

Several previous studies have shown that various imaging modalities, including ultrasound, CT, especially contrast-enhanced CT, ^18^F-FDG PET/CT, and MRI, have the potential to detect HCC with mVI (Lin et al., 2017; Hyun et al., 2018; Hu et al., 2019; Li et al., 2021; Meng et al., 2021). These studies showed that various imaging modalities might have a comparable predictive performance for mVI, whether the morphologic features, metabolic activity features, radiomics analysis, or combination are used. However, it is still unclear which modality is better and unable to completely meet clinical needs to establish risk factor for mVI only by one modality. In this study, we first extracted texture features from ROI in an ^18^F-FDG PET image and selected 30 optimal texture features in our study. Meanwhile, the NDA classification procedure and the neural network classifier on module B11 of the MaZda software package were adopted to construct a radiomics model for predicting HCC with mVI. Our results showed that the radiomics model derived from the image texture feature of axial ^18^F-FDG PET achieved a classification accuracy of 92.6% with an AUC of 0.917 in the training cohort and 79.3% with an AUC of 0.771 in the testing cohort. However, the traditional imaging radiologic features or metabolic activity features, such as quantitative metabolic parameters from PET/CT or quantitative and qualitative MRI parameters, yielded an accuracy range from 58.8% to 73.5% in the training cohort and 58.6%–72.4% in the testing cohort. These results showed that the radiomics model may be much better than the traditional morphologic and metabolic activity features. A recent study showed that a radiomics nomogram based on ^18^F-FDG PET/CT was constructed to predict the mVI status in patients with very-early- and early-stage HCC with an AUC of 0.891 (95% CI: 0.799–0.984) in the training cohort and an AUC of 0.692 (95% CI: 0.497–0.887) in the testing cohort, which also showed that the radiomics model had a strong predictive power in detecting HCC with mVI (Li et al., 2021).

Of course, there are still several remaining significant challenges for radiomics in detecting HCC with mVI, such as replicability, standardization of images and data, and ethical and regulatory considerations (Forghani et al., 2019). In our study, we adopted a method of normalizing image intensities in the range of mean gray-level value ± three standard deviations (SD) to minimize the influence of contrast and brightness variation. In the testing cohort, our radiomics criteria yielded a classification accuracy of 79.3% with an AUC of 0.771 (95% CI: 0.578–0.905), which was lower than that classification accuracy of 92.6% with an AUC of 0.917 (95% CI: 0.824–0.970) in the training cohort. This indicated that the robustness of radiomics was to be improved.

The previous studies have also reported that the quantitative and qualitative parameters of SUVmax, ADC values, hypovascular lesion, and non-smooth tumor margin were associated with mVI status in patients with HCC (Mulé et al., 2020; Zhang et al., 2020). Therefore, in this study, we constructed hybrid criteria combining radiomics criteria and quantitative and qualitative parameters derived from ^18^F-FDG PET/CT and MRI to predict the status of mVI in patients with HCC and achieved better results by significantly increasing AUC, yielded an AUC of 0.996 (95% CI: 0.939 to 1.000; *p* < 0.001) and 0.953 (95% CI: 0.883–1.000) in the training and testing cohorts, respectively. The difference in AUC between hybrid criteria and radiomics criteria showed statistical significance (*p*-values of 0.0165 and 0.0133 in the training and testing cohorts, respectively). Our findings suggest that the utility of the hybrid model combining ^18^F-FDG PET/CT and MRI may improve the preoperative prediction of the status of mVI in HCC, compared to the only utility of the radiomics model based on texture features of ^18^F-FDG PET.

There were several limitations in this study. First, this was a retrospective study with a small sample size, which could influence the robustness and reproducibility of our prediction models. The study samples were only divided into the training and testing cohorts to perform internal validation in this study. There may be a phenomenon of overfitting in the data processing for the AUC significant difference between the radiomics classier training set and the validation set. Therefore, the current study should need further validation with data augmentation and cross-validation in the future. Second, in this study, the HCC ^18^F-FDG PET image textures were extracted only from the two-dimensional largest axial image of each HCC, which may cause loss of the entire tumor heterogeneity information. Therefore, the 3D structures radiomics of the tumor need further study in the future. Third, there was no significant association between the clinical characteristics and the status of mVI in our study (*p* > 0.05). It was different from the previous study by Hyun et al. (2018), which showed that some clinical characteristics of clinical stage, AST, and AFP were significant predictors of mVI. The radiological hybrid model incorporating more clinical, pathological, and prognosis characteristics and radiomics should be considered in future studies.

In conclusion, the hybrid radiological model that incorporates the image texture of the ^18^F-FDG PET signature, quantitative metabolic parameter, and quantitative and qualitative MRI parameters has powerful predictive performance in predicting the status of mVI preoperatively. Thus, such models may facilitate planning clinical treatment and improving survival in selected patients with HCC. Of course, it is warranted to validate the robustness and reproducibility of our prediction models by large-scale multicenter studies in the future.

## Data Availability

The raw data supporting the conclusions of this article will be made available by the authors without undue reservation.
