# Supplementary material for: Role of preoperative prediction of microvascular invasion in hepatocellular carcinoma based on the texture of FDG PET image: A comparison of quantitative metabolic parameters and MRI
Source: Front Physiol. 2022 Aug 12;13:928969. doi: 10.3389/fphys.2022.928969 (PMC9412047; doi:10.3389/fphys.2022.928969)
Supplement: Supplementary file 2 [file Table1.DOCX]

Supplementary Material

**Supplementary Table 1** Radiomic texture features from different categories performed with MaZda software

| Lesion geometry (GEO) | First-order histogram (HIS) | Absolute gradient (GRA) | Run length matrix (RLM) | Co-occurrence matrix (COM) | Autoregressive model (ARM) | Wavelet transform (WAV) |
| --- | --- | --- | --- | --- | --- | --- |
| • Total number of geometric  parameters: 73 | • Mean  • Variance  • Skewness  • Kurtosis  • 1 % percentile  • 10 % percentile  • 50 % percentile  • 90 % percentile  • 99 % percentile | • Mean  • Variance  • Skewness  • Kurtosis  •Percentage of pixels with nonzero gradient | • Run length nonuniformity  • Gray level nonuniformity  • Long run emphasis  • Short run emphasis  • Fraction of image in runs | • Angular second moment  • Contrast  • Correlation  • Sum of squares  • Inverse difference moment  • Sum average  • Sum variance  • Sum entropy  • Entropy  • Difference variance  • Difference entropy | • Teta 1  • Teta 2  • Teta 3  • Teta 4  • Sigma | • Wavelet energy |

**Supplementary Table 2** Parameters of some abdominal MRI sequences

| Sequences Parameters | T1WI | T2WI | DWI |
| --- | --- | --- | --- |
| echo time (TE) (ms) | 1.44 | 70 | 52 |
| repetition time (TR) (ms) | 3.1 | 1610 | 934 |
| field of view (FOV) (mm x mm) | 280 × 305 | 280 × 305 | 280 × 305 |
| matrix | 244 × 186 | 176 × 201 | 100 × 124 |
| slice thickness (mm) | 3 | 7 | 7 |
| slice gap (mm) | 1.5 | 1 | 1 |
| flip angle (degrees) | 10 | 90 | 90 |
| number of excitations (NEXs) | 1 | 2 | 4 |
| number of slices (slices) | 120 | 24 | 48 |
| b values (s/mm2) | - | - | 0, and 800 |

**Supplementary Table 3** optimal features were selected based on Fisher coefficients, minimization of both classification error probability and average correlation coefficients, and mutual information coefficients

| 30 optimal texture features |  |  |
| --- | --- | --- |
| 1 "S(3,0)Contrast" | 11 Teta4 | 21 "S(5,-5)SumOfSqs" |
| 2 "S(5,-5)Contrast" | 12 GeoX | 22 "S(5,-5)DifVarnc" |
| 3 "S(5,5)Entropy" | 13 GeoYo | 23 "S(5,-5)SumAverg" |
| 4 "S(3,-3)Contrast" | 14 "S(5,5)AngScMom" | 24 "S(5,-5)DifEntrp" |
| 5 "S(5,-5)SumEntrp" | 15 GrKurtosis | 25 "S(5,5)Contrast" |
| 6 "S(5,0)Correlat" | 16 GeoAox | 26 GeoW8 |
| 7 "S(3,3)SumOfSqs" | 17 GeoY | 27 "S(4,0)Contrast" |
| 8 "S(3,3)DifVarnc" | 18 Perc.50% | 28 "S(4,-4)SumEntrp" |
| 9 "S(3,0)DifVarnc" | 19 WavEnHH_s-4 | 29 "S(5,-5)Entropy" |
| 10 "S(1,1)Contrast" | 20 "S(4,0)Correlat" | 30 "S(4,4)DifVarnc" |

**Supplementary Figure 1 ROC curve for the performance of quantitative metabolic and diffusion parameters to differentiate HCC with and without mVI.**


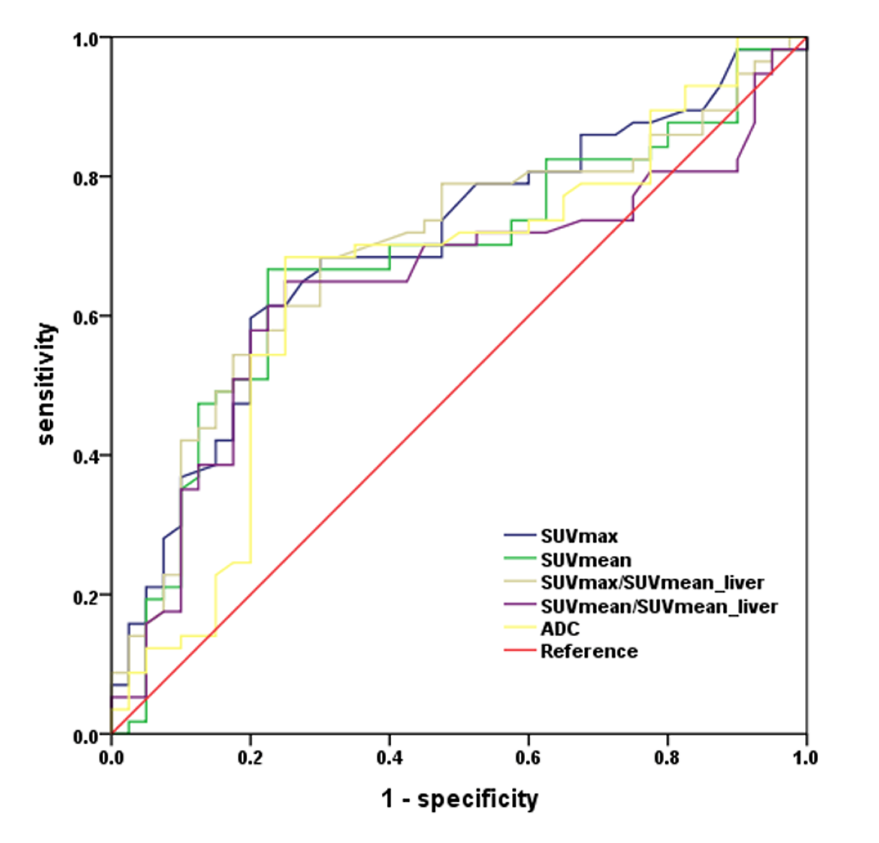


Supplementary Figure 2 Calibration curves of the hybrid model for MVI prediction in the training (a) and test (b) cohorts.


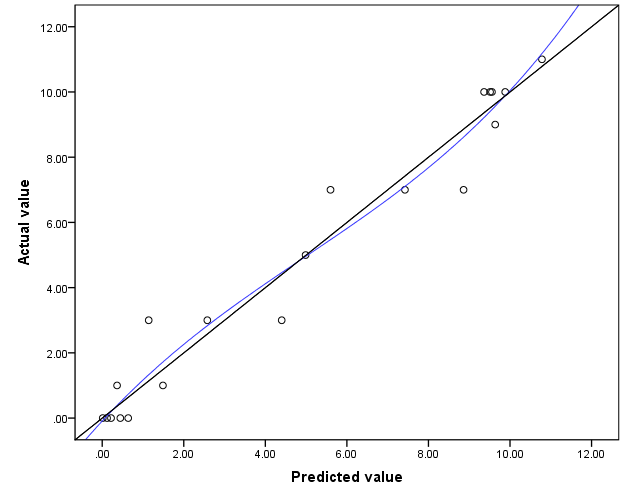
(a)


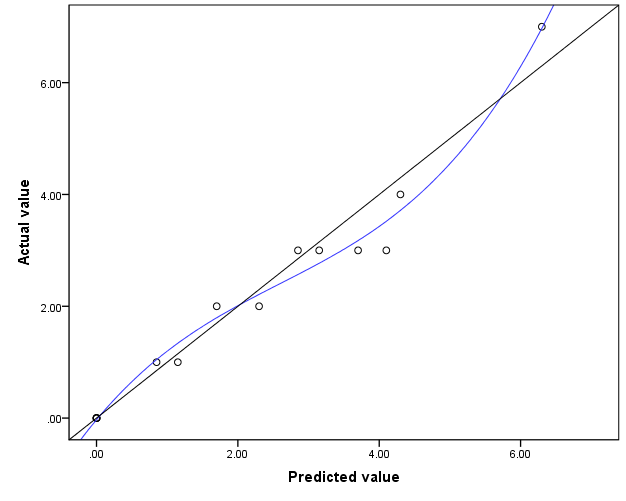
(b)
